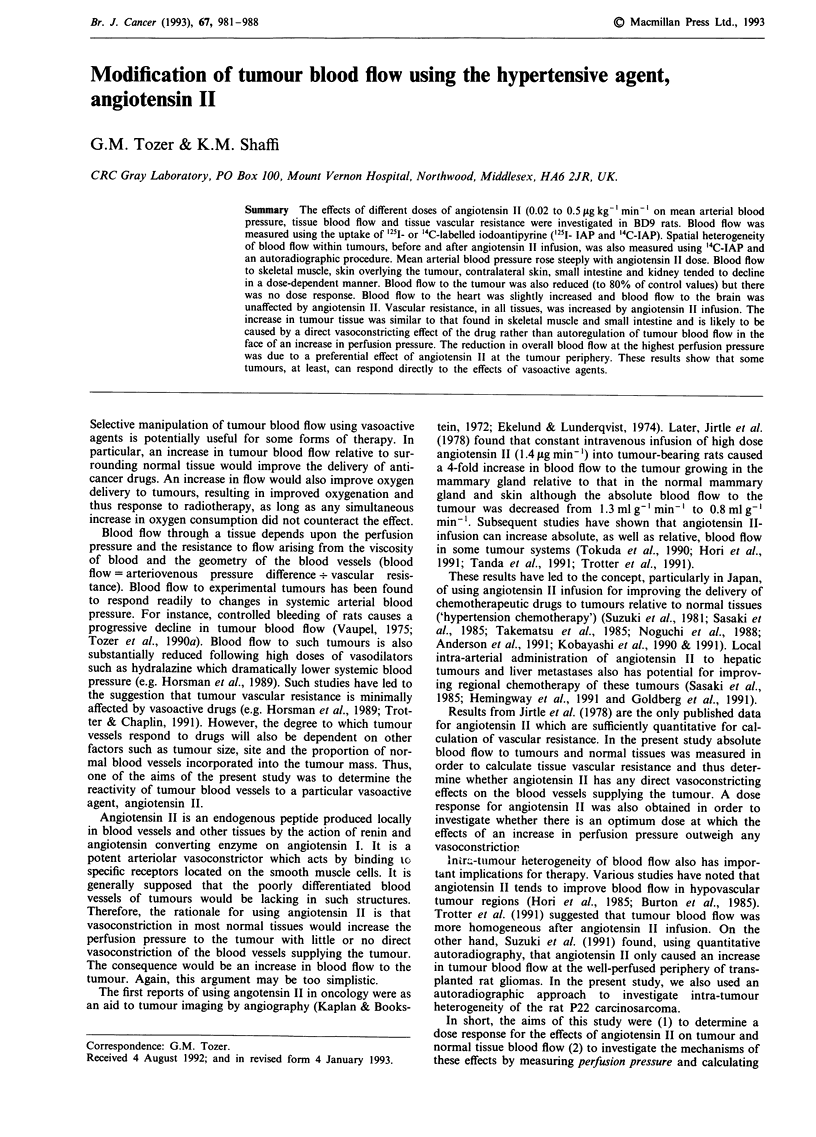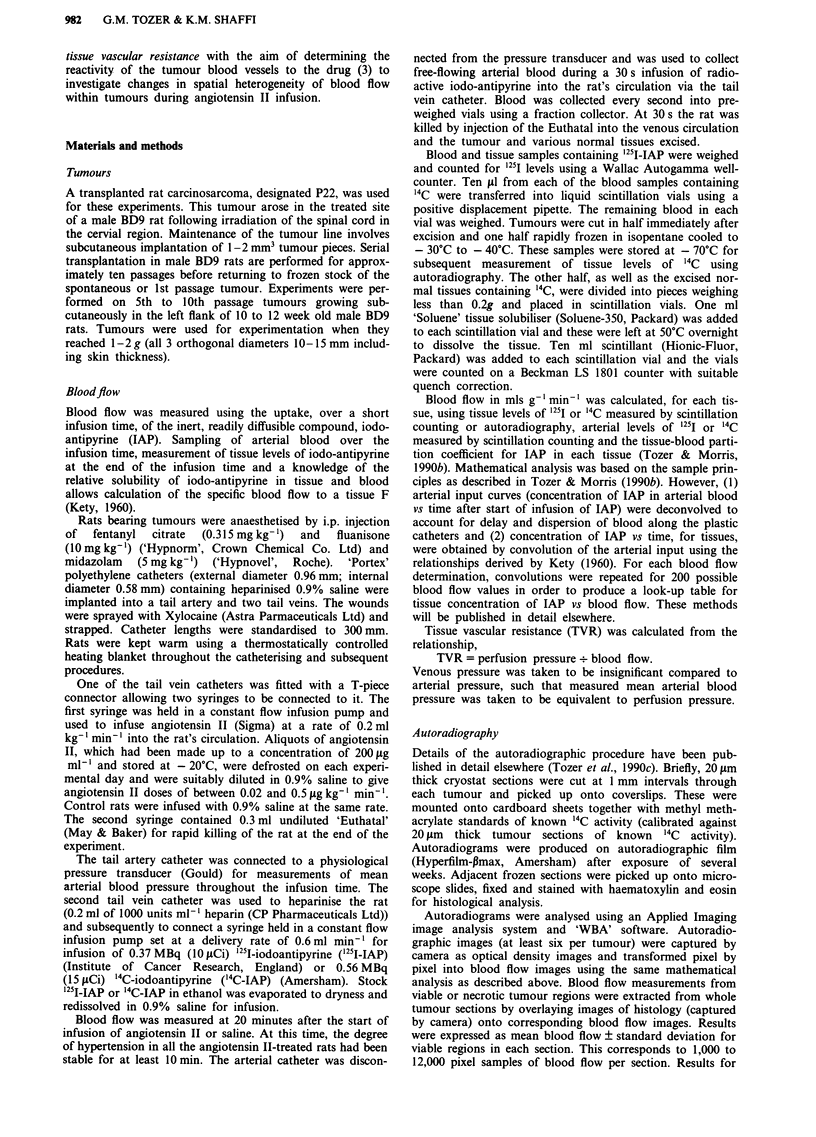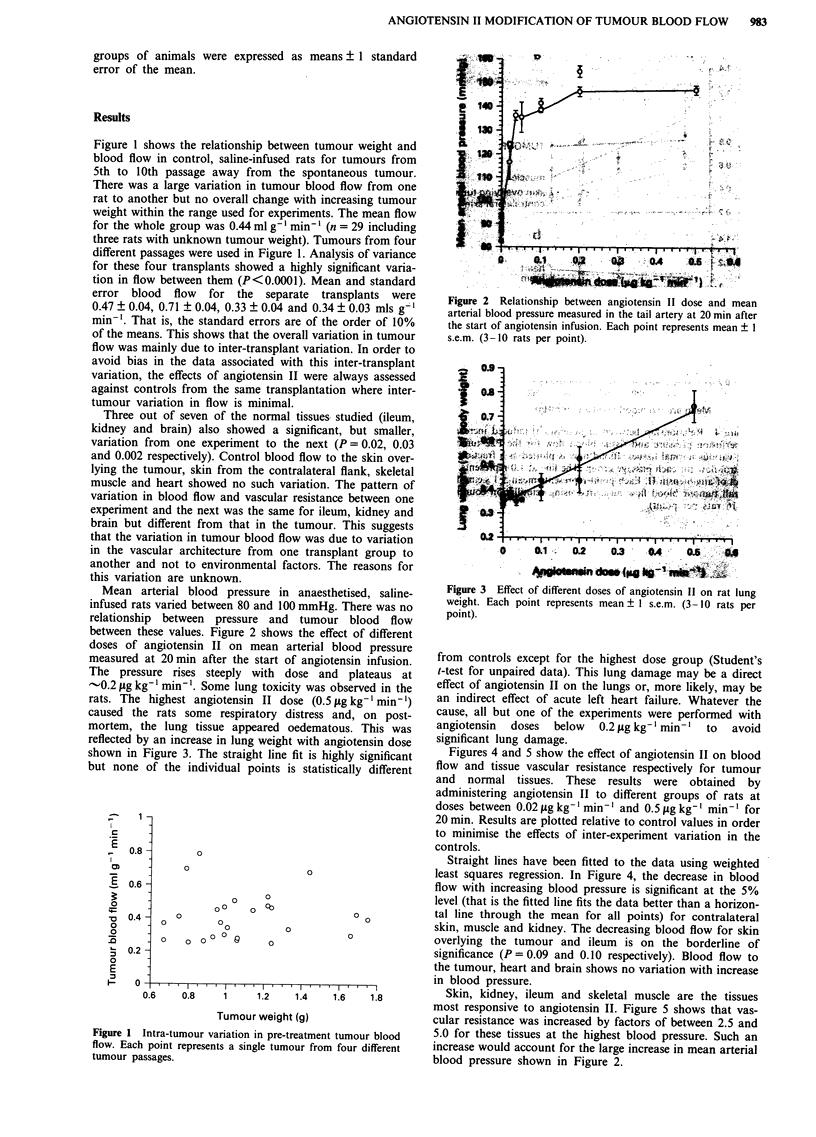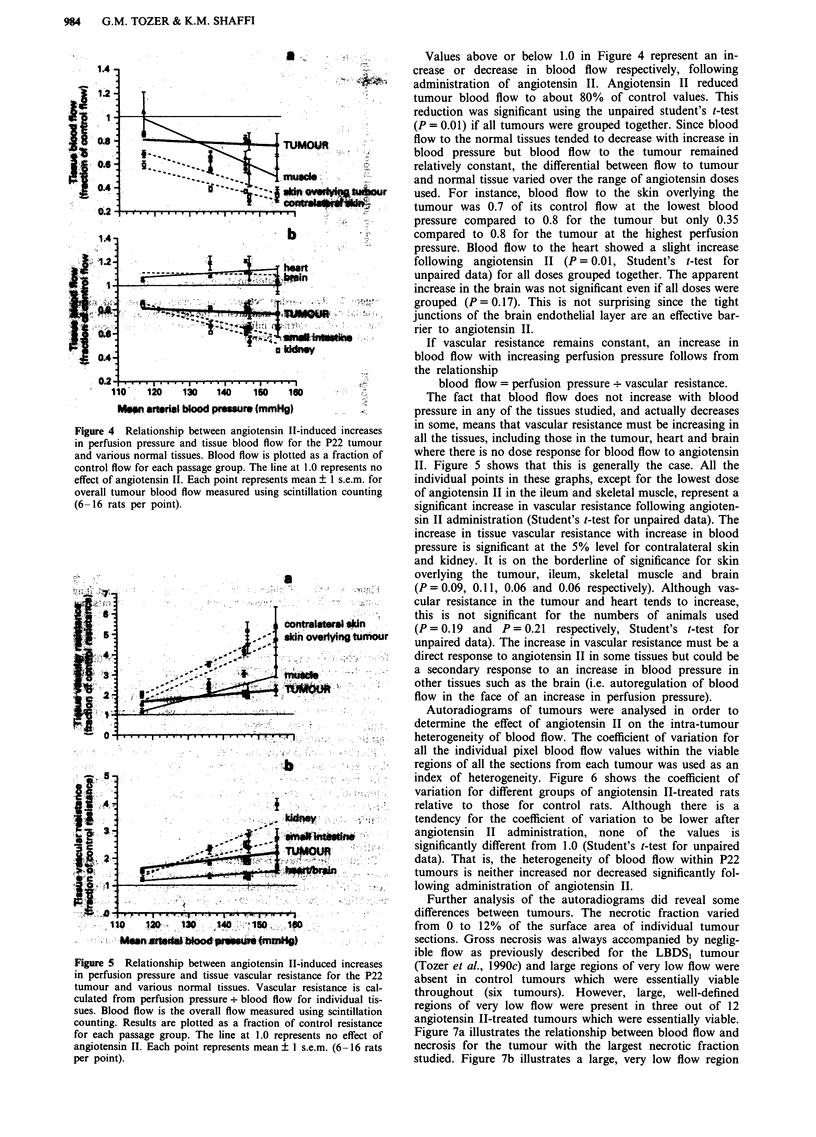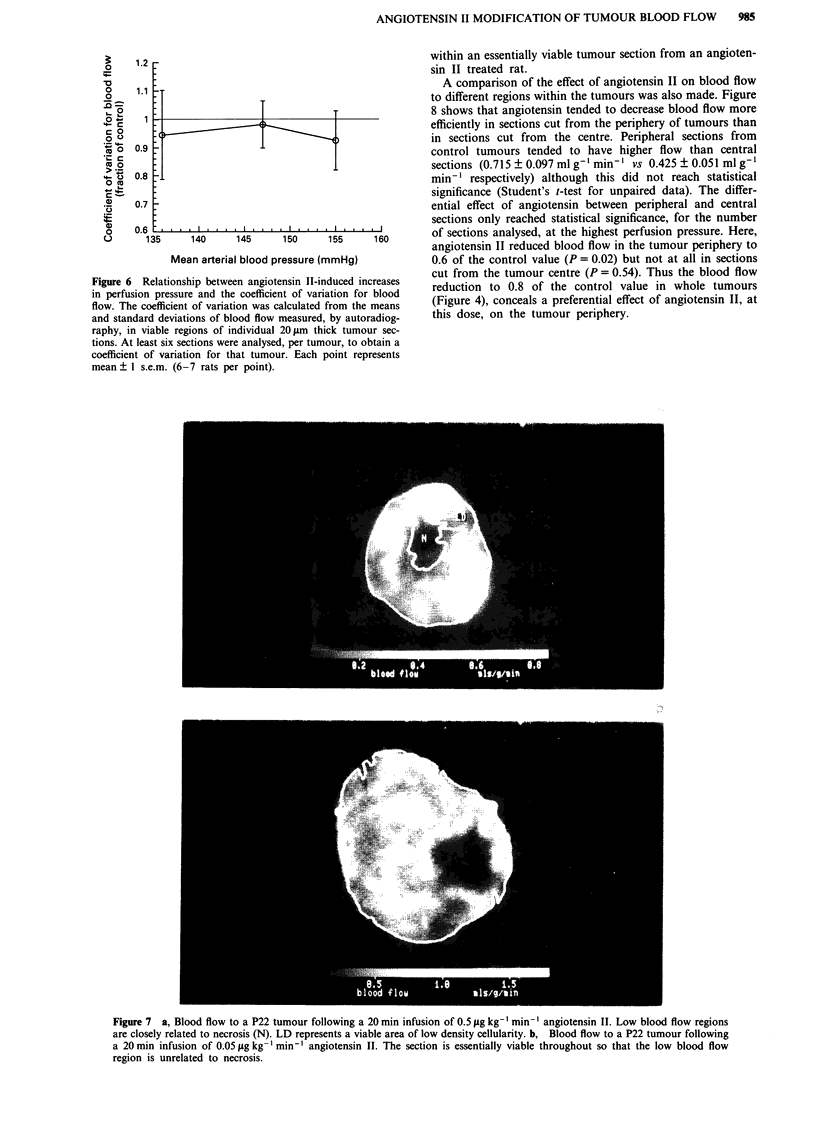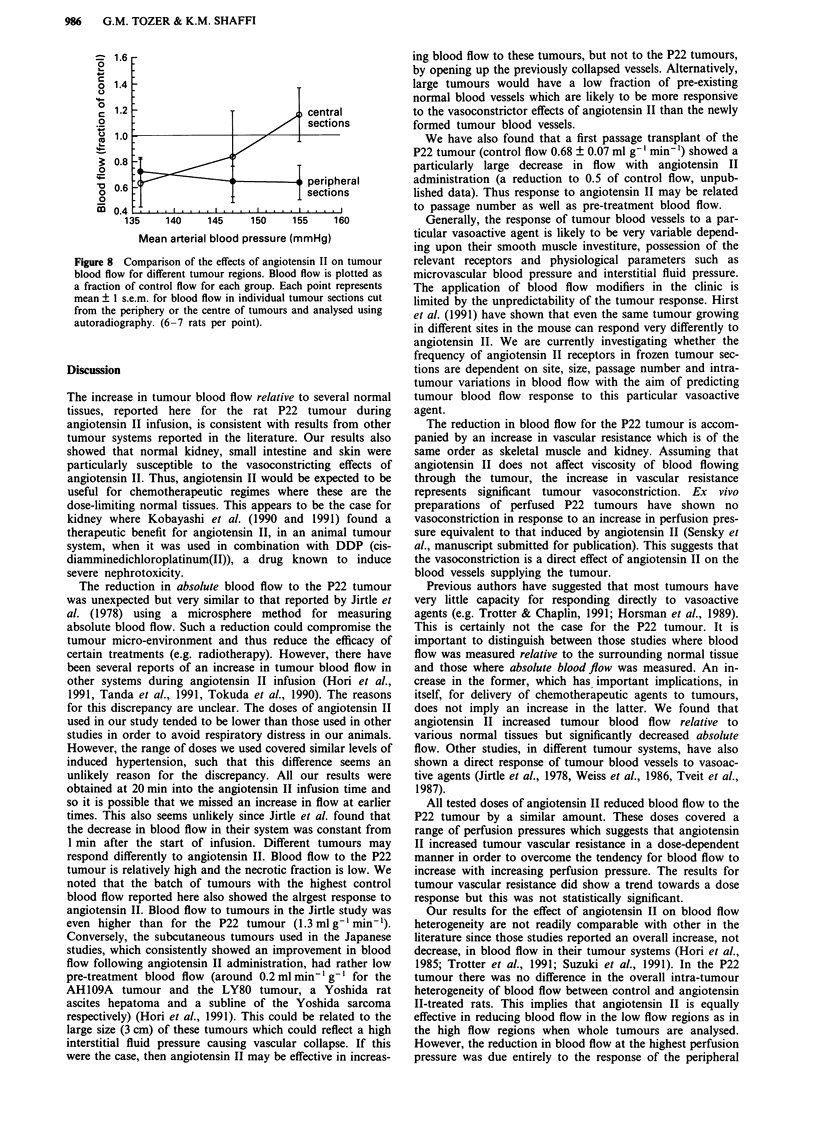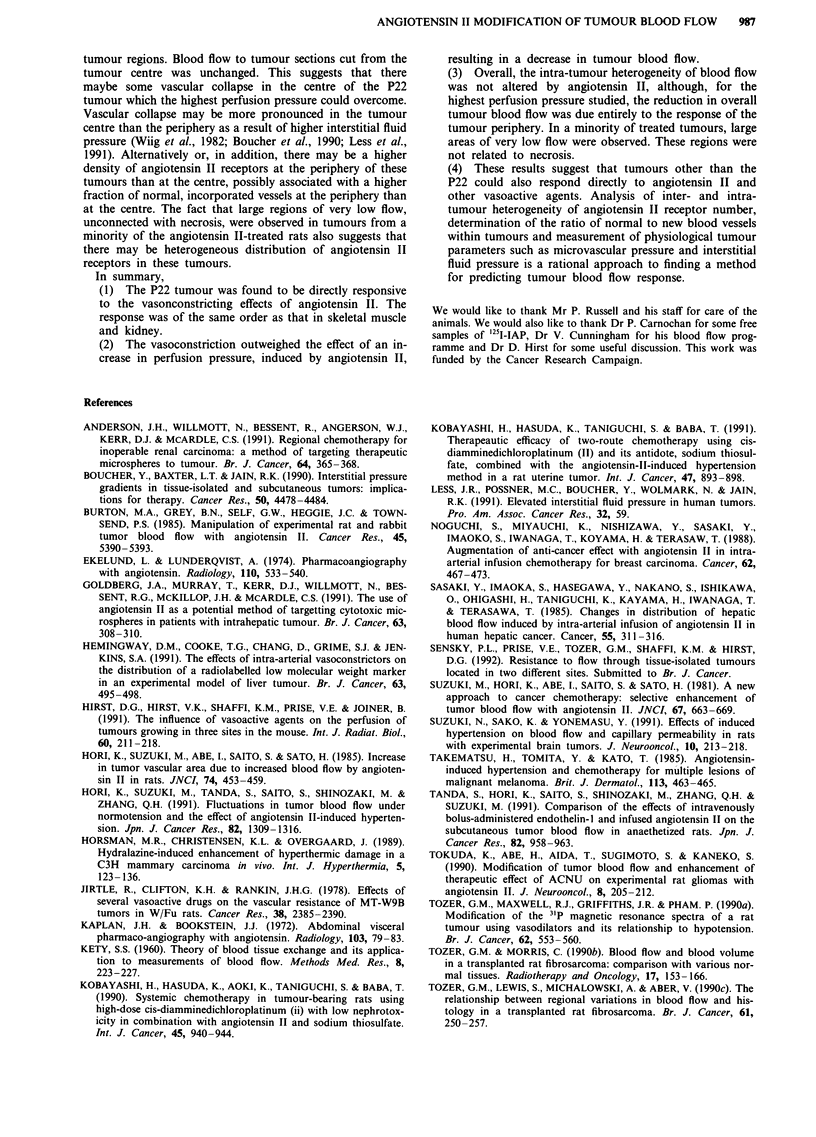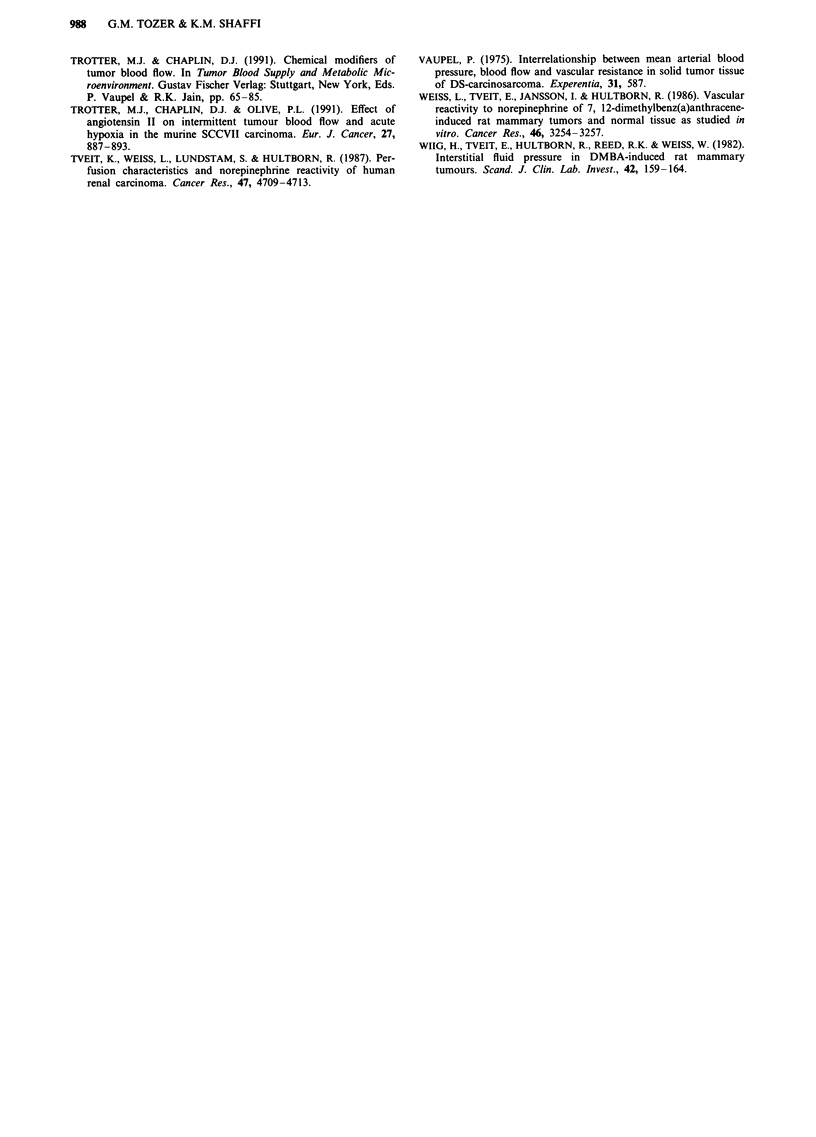# Modification of tumour blood flow using the hypertensive agent, angiotensin II.

**DOI:** 10.1038/bjc.1993.180

**Published:** 1993-05

**Authors:** G. M. Tozer, K. M. Shaffi

**Affiliations:** CRC Gray Laboratory, Mount Vernon Hospital, Northwood, Middlesex, UK.

## Abstract

**Images:**


Br. J. Cancer (1993), 67, 981–988 © Macmillan Press Ltd., 1993

### Modification of tumour blood flow using the hypertensive agent, angiotensin II

G.M. Tozer & K.M. Shaffi

*CRC Gray Laboratory, PO Box 100, Mount Vernon Hospital, Northwood, Middlesex, HA6 2JR, UK.*

**Summary** The effects of different doses of angiotensin II (0.02 to 0.5 $\mu g\ kg^{-1}\ min^{-1}$ on mean arterial blood pressure, tissue blood flow and tissue vascular resistance were investigated in BD9 rats. Blood flow was measured using the uptake of $^{125}I$- or $^{14}C$-labelled iodoantipyrine ($^{125}I$- IAP and $^{14}C$-IAP). Spatial heterogeneity of blood flow within tumours, before and after angiotensin II infusion, was also measured using $^{14}C$-IAP and an autoradiographic procedure. Mean arterial blood pressure rose steeply with angiotensin II dose. Blood flow to skeletal muscle, skin overlying the tumour, contralateral skin, small intestine and kidney tended to decline in a dose-dependent manner. Blood flow to the tumour was also reduced (to 80% of control values) but there was no dose response. Blood flow to the heart was slightly increased and blood flow to the brain was unaffected by angiotensin II. Vascular resistance, in all tissues, was increased by angiotensin II infusion. The increase in tumour tissue was similar to that found in skeletal muscle and small intestine and is likely to be caused by a direct vasoconstricting effect of the drug rather than autoregulation of tumour blood flow in the face of an increase in perfusion pressure. The reduction in overall blood flow at the highest perfusion pressure was due to a preferential effect of angiotensin II at the tumour periphery. These results show that some tumours, at least, can respond directly to the effects of vasoactive agents.

Selective manipulation of tumour blood flow using vasoactive agents is potentially useful for some forms of therapy. In particular, an increase in tumour blood flow relative to surrounding normal tissue would improve the delivery of anti-cancer drugs. An increase in flow would also improve oxygen delivery to tumours, resulting in improved oxygenation and thus response to radiotherapy, as long as any simultaneous increase in oxygen consumption did not counteract the effect.

Blood flow through a tissue depends upon the perfusion pressure and the resistance to flow arising from the viscosity of blood and the geometry of the blood vessels (blood flow = arteriovenous pressure difference ÷ vascular resistance). Blood flow to experimental tumours has been found to respond readily to changes in systemic arterial blood pressure. For instance, controlled bleeding of rats causes a progressive decline in tumour blood flow (Vaupel, 1975; Tozer *et al.*, 1990*a*). Blood flow to such tumours is also substantially reduced following high doses of vasodilators such as hydralazine which dramatically lower systemic blood pressure (e.g. Horsman *et al.*, 1989). Such studies have led to the suggestion that tumour vascular resistance is minimally affected by vasoactive drugs (e.g. Horsman *et al.*, 1989; Trotter & Chaplin, 1991). However, the degree to which tumour vessels respond to drugs will also be dependent on other factors such as tumour size, site and the proportion of normal blood vessels incorporated into the tumour mass. Thus, one of the aims of the present study was to determine the reactivity of tumour blood vessels to a particular vasoactive agent, angiotensin II.

Angiotensin II is an endogenous peptide produced locally in blood vessels and other tissues by the action of renin and angiotensin converting enzyme on angiotensin I. It is a potent arteriolar vasoconstrictor which acts by binding to specific receptors located on the smooth muscle cells. It is generally supposed that the poorly differentiated blood vessels of tumours would be lacking in such structures. Therefore, the rationale for using angiotensin II is that vasoconstriction in most normal tissues would increase the perfusion pressure to the tumour with little or no direct vasoconstriction of the blood vessels supplying the tumour. The consequence would be an increase in blood flow to the tumour. Again, this argument may be too simplistic.

The first reports of using angotensin II in oncology were as an aid to tumour imaging by angiography (Kaplan & Bookstein, 1972; Ekelund & Lunderqvist, 1974). Later, Jirtle *et al.* (1978) found that constant intravenous infusion of high dose angiotensin II (1.4 $\mu g\ min^{-1}$) into tumour-bearing rats caused a 4-fold increase in blood flow to the tumour growing in the mammary gland relative to that in the normal mammary gland and skin although the absolute blood flow to the tumour was decreased from 1.3 $ml\ g^{-1}\ min^{-1}$ to 0.8 $ml\ g^{-1}\ min^{-1}$. Subsequent studies have shown that angiotensin II-infusion can increase absolute, as well as relative, blood flow in some tumour systems (Tokuda *et al.*, 1990; Hori *et al.*, 1991; Tanda *et al.*, 1991; Trotter *et al.*, 1991).

These results have led to the concept, particularly in Japan, of using angiotensin II infusion for improving the delivery of chemotherapeutic drugs to tumours relative to normal tissues ('hypertension chemotherapy') (Suzuki *et al.*, 1981; Sasaki *et al.*, 1985; Takematsu *et al.*, 1985; Noguchi *et al.*, 1988; Anderson *et al.*, 1991; Kobayashi *et al.*, 1990 & 1991). Local intra-arterial administration of angiotensin II to hepatic tumours and liver metastases also has potential for improving regional chemotherapy of these tumours (Sasaki *et al.*, 1985; Hemingway *et al.*, 1991 and Goldberg *et al.*, 1991).

Results from Jirtle *et al.* (1978) are the only published data for angiotensin II which are sufficiently quantitative for calculation of vascular resistance. In the present study absolute blood flow to tumours and normal tissues was measured in order to calculate tissue vascular resistance and thus determine whether angiotensin II has any direct vasoconstricting effects on the blood vessels supplying the tumour. A dose response for angiotensin II was also obtained in order to investigate whether there is an optimum dose at which the effects of an increase in perfusion pressure outweigh any vasoconstriction.

Intra-tumour heterogeneity of blood flow also has important implications for therapy. Various studies have noted that angiotensin II tends to improve blood flow in hypovascular tumour regions (Hori *et al.*, 1985; Burton *et al.*, 1985). Trotter *et al.* (1991) suggested that tumour blood flow was more homogeneous after angiotensin II infusion. On the other hand, Suzuki *et al.* (1991) found, using quantitative autoradiography, that angiotensin II only caused an increase in tumour blood flow at the well-perfused periphery of transplanted rat gliomas. In the present study, we also used an autoradiographic approach to investigate intra-tumour heterogeneity of the rat P22 carcinosarcoma.

In short, the aims of this study were (1) to determine a dose response for the effects of angiotensin II on tumour and normal tissue blood flow (2) to investigate the mechanisms of these effects by measuring *perfusion pressure* and calculating

Correspondence: G.M. Tozer.
Received 4 August 1992; and in revised form 4 January 1993.

*tissue vascular resistance* with the aim of determining the reactivity of the tumour blood vessels to the drug (3) to investigate changes in spatial heterogeneity of blood flow within tumours during angiotensin II infusion.

### Materials and methods

### *Tumours*

A transplanted rat carcinosarcoma, designated P22, was used for these experiments. This tumour arose in the treated site of a male BD9 rat following irradiation of the spinal cord in the cervial region. Maintenance of the tumour line involves subcutaneous implantation of 1–2 $mm^3$ tumour pieces. Serial transplantation in male BD9 rats are performed for approximately ten passages before returning to frozen stock of the spontaneous or 1st passage tumour. Experiments were performed on 5th to 10th passage tumours growing subcutaneously in the left flank of 10 to 12 week old male BD9 rats. Tumours were used for experimentation when they reached 1–2 *g* (all 3 orthogonal diameters 10–15 mm including skin thickness).

### *Blood flow*

Blood flow was measured using the uptake, over a short infusion time, of the inert, readily diffusible compound, iodo-antipyrine (IAP). Sampling of arterial blood over the infusion time, measurement of tissue levels of iodo-antipyrine at the end of the infusion time and a knowledge of the relative solubility of iodo-antipyrine in tissue and blood allows calculation of the specific blood flow to a tissue F (Kety, 1960).

Rats bearing tumours were anaesthetised by i.p. injection of fentanyl citrate (0.315 mg $kg^{-1}$) and fluanisone (10 mg $kg^{-1}$) ('Hypnorm', Crown Chemical Co. Ltd) and midazolam (5 mg $kg^{-1}$) ('Hypnovel', Roche). 'Portex' polyethylene catheters (external diameter 0.96 mm; internal diameter 0.58 mm) containing heparinised 0.9% saline were implanted into a tail artery and two tail veins. The wounds were sprayed with Xylocaine (Astra Parmaceuticals Ltd) and strapped. Catheter lengths were standardised to 300 mm. Rats were kept warm using a thermostatically controlled heating blanket throughout the catheterising and subsequent procedures.

One of the tail vein catheters was fitted with a T-piece connector allowing two syringes to be connected to it. The first syringe was held in a constant flow infusion pump and used to infuse angiotensin II (Sigma) at a rate of 0.2 ml $kg^{-1}$ $min^{-1}$ into the rat's circulation. Aliquots of angiotensin II, which had been made up to a concentration of 200 $\mu g$ $ml^{-1}$ and stored at −20°C, were defrosted on each experimental day and were suitably diluted in 0.9% saline to give angiotensin II doses of between 0.02 and 0.5 $\mu g$ $kg^{-1}$ $min^{-1}$. Control rats were infused with 0.9% saline at the same rate. The second syringe contained 0.3 ml undiluted 'Euthatal' (May & Baker) for rapid killing of the rat at the end of the experiment.

The tail artery catheter was connected to a physiological pressure transducer (Gould) for measurements of mean arterial blood pressure throughout the infusion time. The second tail vein catheter was used to heparinise the rat (0.2 ml of 1000 units $ml^{-1}$ heparin (CP Pharmaceuticals Ltd)) and subsequently to connect a syringe held in a constant flow infusion pump set at a delivery rate of 0.6 ml $min^{-1}$ for infusion of 0.37 MBq (10 $\mu Ci$) $^{125}I$-iodoantipyrine ($^{125}I$-IAP) (Institute of Cancer Research, England) or 0.56 MBq (15 $\mu Ci$) $^{14}C$-iodoantipyrine ($^{14}C$-IAP) (Amersham). Stock $^{125}I$-IAP or $^{14}C$-IAP in ethanol was evaporated to dryness and redissolved in 0.9% saline for infusion.

Blood flow was measured at 20 minutes after the start of infusion of angiotensin II or saline. At this time, the degree of hypertension in all the angiotensin II-treated rats had been stable for at least 10 min. The arterial catheter was disconnected from the pressure transducer and was used to collect free-flowing arterial blood during a 30 s infusion of radioactive iodo-antipyrine into the rat's circulation via the tail vein catheter. Blood was collected every second into preweighed vials using a fraction collector. At 30 s the rat was killed by injection of the Euthatal into the venous circulation and the tumour and various normal tissues excised.

Blood and tissue samples containing $^{125}I$-IAP were weighed and counted for $^{125}I$ levels using a Wallac Autogamma well-counter. Ten $\mu l$ from each of the blood samples containing $^{14}C$ were transferred into liquid scintillation vials using a positive displacement pipette. The remaining blood in each vial was weighed. Tumours were cut in half immediately after excision and one half rapidly frozen in isopentane cooled to −30°C to −40°C. These samples were stored at −70°C for subsequent measurement of tissue levels of $^{14}C$ using autoradiography. The other half, as well as the excised normal tissues containing $^{14}C$, were divided into pieces weighing less than 0.2*g* and placed in scintillation vials. One ml 'Soluene' tissue solubiliser (Soluene-350, Packard) was added to each scintillation vial and these were left at 50°C overnight to dissolve the tissue. Ten ml scintillant (Hionic-Fluor, Packard) was added to each scintillation vial and the vials were counted on a Beckman LS 1801 counter with suitable quench correction.

Blood flow in mls $g^{-1}$ $min^{-1}$ was calculated, for each tissue, using tissue levels of $^{125}I$ or $^{14}C$ measured by scintillation counting or autoradiography, arterial levels of $^{125}I$ or $^{14}C$ measured by scintillation counting and the tissue-blood partition coefficient for IAP in each tissue (Tozer & Morris, 1990*b*). Mathematical analysis was based on the sample principles as described in Tozer & Morris (1990*b*). However, (1) arterial input curves (concentration of IAP in arterial blood *vs* time after start of infusion of IAP) were deconvolved to account for delay and dispersion of blood along the plastic catheters and (2) concentration of IAP *vs* time, for tissues, were obtained by convolution of the arterial input using the relationships derived by Kety (1960). For each blood flow determination, convolutions were repeated for 200 possible blood flow values in order to produce a look-up table for tissue concentration of IAP *vs* blood flow. These methods will be published in detail elsewhere.

Tissue vascular resistance (TVR) was calculated from the relationship,

$$\text{TVR} = \text{perfusion pressure} \div \text{blood flow}.$$

Venous pressure was taken to be insignificant compared to arterial pressure, such that measured mean arterial blood pressure was taken to be equivalent to perfusion pressure.

### *Autoradiography*

Details of the autoradiographic procedure have been published in detail elsewhere (Tozer *et al.*, 1990*c*). Briefly, 20 $\mu m$ thick cryostat sections were cut at 1 mm intervals through each tumour and picked up onto coverslips. These were mounted onto cardboard sheets together with methyl methacrylate standards of known $^{14}C$ activity (calibrated against 20 $\mu m$ thick tumour sections of known $^{14}C$ activity). Autoradiograms were produced on autoradiographic film (Hyperfilm-$\beta$max, Amersham) after exposure of several weeks. Adjacent frozen sections were picked up onto microscope slides, fixed and stained with haematoxylin and eosin for histological analysis.

Autoradiograms were analysed using an Applied Imaging image analysis system and 'WBA' software. Autoradiographic images (at least six per tumour) were captured by camera as optical density images and transformed pixel by pixel into blood flow images using the same mathematical analysis as described above. Blood flow measurements from viable or necrotic tumour regions were extracted from whole tumour sections by overlaying images of histology (captured by camera) onto corresponding blood flow images. Results were expressed as mean blood flow ± standard deviation for viable regions in each section. This corresponds to 1,000 to 12,000 pixel samples of blood flow per section. Results for groups of animals were expressed as means ± 1 standard error of the mean.

### Results

Figure 1 shows the relationship between tumour weight and blood flow in control, saline-infused rats for tumours from 5th to 10th passage away from the spontaneous tumour. There was a large variation in tumour blood flow from one rat to another but no overall change with increasing tumour weight within the range used for experiments. The mean flow for the whole group was 0.44 ml $g^{-1}$ $min^{-1}$ ($n = 29$ including three rats with unknown tumour weight). Tumours from four different passages were used in Figure 1. Analysis of variance for these four transplants showed a highly significant variation in flow between them ($P < 0.0001$). Mean and standard error blood flow for the separate transplants were 0.47 ± 0.04, 0.71 ± 0.04, 0.33 ± 0.04 and 0.34 ± 0.03 mls $g^{-1}$ $min^{-1}$. That is, the standard errors are of the order of 10% of the means. This shows that the overall variation in tumour flow was mainly due to inter-transplant variation. In order to avoid bias in the data associated with this inter-transplant variation, the effects of angiotensin II were always assessed against controls from the same transplantation where inter-tumour variation in flow is minimal.

Three out of seven of the normal tissues studied (ileum, kidney and brain) also showed a significant, but smaller, variation from one experiment to the next ($P = 0.02$, 0.03 and 0.002 respectively). Control blood flow to the skin overlying the tumour, skin from the contralateral flank, skeletal muscle and heart showed no such variation. The pattern of variation in blood flow and vascular resistance between one experiment and the next was the same for ileum, kidney and brain but different from that in the tumour. This suggests that the variation in tumour blood flow was due to variation in the vascular architecture from one transplant group to another and not to environmental factors. The reasons for this variation are unknown.

Mean arterial blood pressure in anaesthetised, saline-infused rats varied between 80 and 100 mmHg. There was no relationship between pressure and tumour blood flow between these values. Figure 2 shows the effect of different doses of angiotensin II on mean arterial blood pressure measured at 20 min after the start of angiotensin infusion. The pressure rises steeply with dose and plateaus at ~0.2 μg $kg^{-1}$ $min^{-1}$. Some lung toxicity was observed in the rats. The highest angiotensin II dose (0.5 μg $kg^{-1}$ $min^{-1}$) caused the rats some respiratory distress and, on post-mortem, the lung tissue appeared oedematous. This was reflected by an increase in lung weight with angiotensin dose shown in Figure 3. The straight line fit is highly significant but none of the individual points is statistically different from controls except for the highest dose group (Student's $t$-test for unpaired data). This lung damage may be a direct effect of angiotensin II on the lungs or, more likely, may be an indirect effect of acute left heart failure. Whatever the cause, all but one of the experiments were performed with angiotensin doses below 0.2 μg $kg^{-1}$ $min^{-1}$ to avoid significant lung damage.

**Figure 1** Intra-tumour variation in pre-treatment tumour blood flow. Each point represents a single tumour from four different tumour passages.

**Figure 2** Relationship between angiotensin II dose and mean arterial blood pressure measured in the tail artery at 20 min after the start of angiotensin infusion. Each point represents mean ± 1 s.e.m. (3–10 rats per point).

**Figure 3** Effect of different doses of angiotensin II on rat lung weight. Each point represents mean ± 1 s.e.m. (3–10 rats per point).

Figures 4 and 5 show the effect of angiotensin II on blood flow and tissue vascular resistance respectively for tumour and normal tissues. These results were obtained by administering angiotensin II to different groups of rats at doses between 0.02 μg $kg^{-1}$ $min^{-1}$ and 0.5 μg $kg^{-1}$ $min^{-1}$ for 20 min. Results are plotted relative to control values in order to minimise the effects of inter-experiment variation in the controls.

Straight lines have been fitted to the data using weighted least squares regression. In Figure 4, the decrease in blood flow with increasing blood pressure is significant at the 5% level (that is the fitted line fits the data better than a horizontal line through the mean for all points) for contralateral skin, muscle and kidney. The decreasing blood flow for skin overlying the tumour and ileum is on the borderline of significance ($P = 0.09$ and 0.10 respectively). Blood flow to the tumour, heart and brain shows no variation with increase in blood pressure.

Skin, kidney, ileum and skeletal muscle are the tissues most responsive to angiotensin II. Figure 5 shows that vascular resistance was increased by factors of between 2.5 and 5.0 for these tissues at the highest blood pressure. Such an increase would account for the large increase in mean arterial blood pressure shown in Figure 2.

**Figure 4** Relationship between angiotensin II-induced increases in perfusion pressure and tissue blood flow for the P22 tumour and various normal tissues. Blood flow is plotted as a fraction of control flow for each passage group. The line at 1.0 represents no effect of angiotensin II. Each point represents mean ± 1 s.e.m. for overall tumour blood flow measured using scintillation counting (6–16 rats per point).

**Figure 5** Relationship between angiotensin II-induced increases in perfusion pressure and tissue vascular resistance for the P22 tumour and various normal tissues. Vascular resistance is calculated from perfusion pressure ÷ blood flow for individual tissues. Blood flow is the overall flow measured using scintillation counting. Results are plotted as a fraction of control resistance for each passage group. The line at 1.0 represents no effect of angiotensin II. Each point represents mean ± 1 s.e.m. (6–16 rats per point).

Values above or below 1.0 in Figure 4 represent an increase or decrease in blood flow respectively, following administration of angiotensin II. Angiotensin II reduced tumour blood flow to about 80% of control values. This reduction was significant using the unpaired student's *t*-test ($P = 0.01$) if all tumours were grouped together. Since blood flow to the normal tissues tended to decrease with increase in blood pressure but blood flow to the tumour remained relatively constant, the differential between flow to tumour and normal tissue varied over the range of angiotensin doses used. For instance, blood flow to the skin overlying the tumour was 0.7 of its control flow at the lowest blood pressure compared to 0.8 for the tumour but only 0.35 compared to 0.8 for the tumour at the highest perfusion pressure. Blood flow to the heart showed a slight increase following angiotensin II ($P = 0.01$, Student's *t*-test for unpaired data) for all doses grouped together. The apparent increase in the brain was not significant even if all doses were grouped ($P = 0.17$). This is not surprising since the tight junctions of the brain endothelial layer are an effective barrier to angiotensin II.

If vascular resistance remains constant, an increase in blood flow with increasing perfusion pressure follows from the relationship

$$\text{blood flow} = \text{perfusion pressure} \div \text{vascular resistance.}$$

The fact that blood flow does not increase with blood pressure in any of the tissues studied, and actually decreases in some, means that vascular resistance must be increasing in all the tissues, including those in the tumour, heart and brain where there is no dose response for blood flow to angiotensin II. Figure 5 shows that this is generally the case. All the individual points in these graphs, except for the lowest dose of angiotensin II in the ileum and skeletal muscle, represent a significant increase in vascular resistance following angiotensin II administration (Student's *t*-test for unpaired data). The increase in tissue vascular resistance with increase in blood pressure is significant at the 5% level for contralateral skin and kidney. It is on the borderline of significance for skin overlying the tumour, ileum, skeletal muscle and brain ($P = 0.09$, 0.11, 0.06 and 0.06 respectively). Although vascular resistance in the tumour and heart tends to increase, this is not significant for the numbers of animals used ($P = 0.19$ and $P = 0.21$ respectively, Student's *t*-test for unpaired data). The increase in vascular resistance must be a direct response to angiotensin II in some tissues but could be a secondary response to an increase in blood pressure in other tissues such as the brain (i.e. autoregulation of blood flow in the face of an increase in perfusion pressure).

Autoradiograms of tumours were analysed in order to determine the effect of angiotensin II on the intra-tumour heterogeneity of blood flow. The coefficient of variation for all the individual pixel blood flow values within the viable regions of all the sections from each tumour was used as an index of heterogeneity. Figure 6 shows the coefficient of variation for different groups of angiotensin II-treated rats relative to those for control rats. Although there is a tendency for the coefficient of variation to be lower after angiotensin II administration, none of the values is significantly different from 1.0 (Student's *t*-test for unpaired data). That is, the heterogeneity of blood flow within P22 tumours is neither increased nor decreased significantly following administration of angiotensin II.

Further analysis of the autoradiograms did reveal some differences between tumours. The necrotic fraction varied from 0 to 12% of the surface area of individual tumour sections. Gross necrosis was always accompanied by negligible flow as previously described for the $LBDS_1$ tumour (Tozer *et al.*, 1990*c*) and large regions of very low flow were absent in control tumours which were essentially viable throughout (six tumours). However, large, well-defined regions of very low flow were present in three out of 12 angiotensin II-treated tumours which were essentially viable. Figure 7a illustrates the relationship between blood flow and necrosis for the tumour with the largest necrotic fraction studied. Figure 7b illustrates a large, very low flow region

**Figure 6** Relationship between angiotensin II-induced increases in perfusion pressure and the coefficient of variation for blood flow. The coefficient of variation was calculated from the means and standard deviations of blood flow measured, by autoradiography, in viable regions of individual 20 $\mu$m thick tumour sections. At least six sections were analysed, per tumour, to obtain a coefficient of variation for that tumour. Each point represents mean ± 1 s.e.m. (6–7 rats per point).

within an essentially viable tumour section from an angiotensin II treated rat.

A comparison of the effect of angiotensin II on blood flow to different regions within the tumours was also made. Figure 8 shows that angiotensin tended to decrease blood flow more efficiently in sections cut from the periphery of tumours than in sections cut from the centre. Peripheral sections from control tumours tended to have higher flow than central sections (0.715 ± 0.097 ml $g^{-1}$ $min^{-1}$ *vs* 0.425 ± 0.051 ml $g^{-1}$ $min^{-1}$ respectively) although this did not reach statistical significance (Student's *t*-test for unpaired data). The differential effect of angiotensin between peripheral and central sections only reached statistical significance, for the number of sections analysed, at the highest perfusion pressure. Here, angiotensin II reduced blood flow in the tumour periphery to 0.6 of the control value ($P = 0.02$) but not at all in sections cut from the tumour centre ($P = 0.54$). Thus the blood flow reduction to 0.8 of the control value in whole tumours (Figure 4), conceals a preferential effect of angiotensin II, at this dose, on the tumour periphery.

**Figure 7** **a**, Blood flow to a P22 tumour following a 20 min infusion of 0.5 $\mu$g $kg^{-1}$ $min^{-1}$ angiotensin II. Low blood flow regions are closely related to necrosis (N). LD represents a viable area of low density cellularity. **b**, Blood flow to a P22 tumour following a 20 min infusion of 0.05 $\mu$g $kg^{-1}$ $min^{-1}$ angiotensin II. The section is essentially viable throughout so that the low blood flow region is unrelated to necrosis.

**Figure 8** Comparison of the effects of angiotensin II on tumour blood flow for different tumour regions. Blood flow is plotted as a fraction of control flow for each group. Each point represents mean ± 1 s.e.m. for blood flow in individual tumour sections cut from the periphery or the centre of tumours and analysed using autoradiography. (6–7 rats per point).

### Discussion

The increase in tumour blood flow *relative* to several normal tissues, reported here for the rat P22 tumour during angiotensin II infusion, is consistent with results from other tumour systems reported in the literature. Our results also showed that normal kidney, small intestine and skin were particularly susceptible to the vasoconstricting effects of angiotensin II. Thus, angiotensin II would be expected to be useful for chemotherapeutic regimes where these are the dose-limiting normal tissues. This appears to be the case for kidney where Kobayashi *et al.* (1990 and 1991) found a therapeutic benefit for angiotensin II, in an animal tumour system, when it was used in combination with DDP (cis-diamminedichloroplatinum(II)), a drug known to induce severe nephrotoxicity.

The reduction in *absolute* blood flow to the P22 tumour was unexpected but very similar to that reported by Jirtle *et al.* (1978) using a microsphere method for measuring absolute blood flow. Such a reduction could compromise the tumour micro-environment and thus reduce the efficacy of certain treatments (e.g. radiotherapy). However, there have been several reports of an increase in tumour blood flow in other systems during angiotensin II infusion (Hori *et al.*, 1991, Tanda *et al.*, 1991, Tokuda *et al.*, 1990). The reasons for this discrepancy are unclear. The doses of angiotensin II used in our study tended to be lower than those used in other studies in order to avoid respiratory distress in our animals. However, the range of doses we used covered similar levels of induced hypertension, such that this difference seems an unlikely reason for the discrepancy. All our results were obtained at 20 min into the angiotensin II infusion time and so it is possible that we missed an increase in flow at earlier times. This also seems unlikely since Jirtle *et al.* found that the decrease in blood flow in their system was constant from 1 min after the start of infusion. Different tumours may respond differently to angiotensin II. Blood flow to the P22 tumour is relatively high and the necrotic fraction is low. We noted that the batch of tumours with the highest control blood flow reported here also showed the alrgest response to angiotensin II. Blood flow to tumours in the Jirtle study was even higher than for the P22 tumour (1.3 ml $g^{-1}$ $min^{-1}$). Conversely, the subcutaneous tumours used in the Japanese studies, which consistently showed an improvement in blood flow following angiotensin II administration, had rather low pre-treatment blood flow (around 0.2 ml $min^{-1}$ $g^{-1}$ for the AH109A tumour and the LY80 tumour, a Yoshida rat ascites hepatoma and a subline of the Yoshida sarcoma respectively) (Hori *et al.*, 1991). This could be related to the large size (3 cm) of these tumours which could reflect a high interstitial fluid pressure causing vascular collapse. If this were the case, then angiotensin II may be effective in increasing blood flow to these tumours, but not to the P22 tumours, by opening up the previously collapsed vessels. Alternatively, large tumours would have a low fraction of pre-existing normal blood vessels which are likely to be more responsive to the vasoconstrictor effects of angiotensin II than the newly formed tumour blood vessels.

We have also found that a first passage transplant of the P22 tumour (control flow 0.68 ± 0.07 ml $g^{-1}$ $min^{-1}$) showed a particularly large decrease in flow with angiotensin II administration (a reduction to 0.5 of control flow, unpublished data). Thus response to angiotensin II may be related to passage number as well as pre-treatment blood flow.

Generally, the response of tumour blood vessels to a particular vasoactive agent is likely to be very variable depending upon their smooth muscle investiture, possession of the relevant receptors and physiological parameters such as microvascular blood pressure and interstitial fluid pressure. The application of blood flow modifiers in the clinic is limited by the unpredictability of the tumour response. Hirst *et al.* (1991) have shown that even the same tumour growing in different sites in the mouse can respond very differently to angiotensin II. We are currently investigating whether the frequency of angiotensin II receptors in frozen tumour sections are dependent on site, size, passage number and intra-tumour variations in blood flow with the aim of predicting tumour blood flow response to this particular vasoactive agent.

The reduction in blood flow for the P22 tumour is accompanied by an increase in vascular resistance which is of the same order as skeletal muscle and kidney. Assuming that angiotensin II does not affect viscosity of blood flowing through the tumour, the increase in vascular resistance represents significant tumour vasoconstriction. *Ex vivo* preparations of perfused P22 tumours have shown no vasoconstriction in response to an increase in perfusion pressure equivalent to that induced by angiotensin II (Sensky *et al.*, manuscript submitted for publication). This suggests that the vasoconstriction is a direct effect of angiotensin II on the blood vessels supplying the tumour.

Previous authors have suggested that most tumours have very little capacity for responding directly to vasoactive agents (e.g. Trotter & Chaplin, 1991; Horsman *et al.*, 1989). This is certainly not the case for the P22 tumour. It is important to distinguish between those studies where blood flow was measured *relative* to the surrounding normal tissue and those where *absolute blood flow* was measured. An increase in the former, which has important implications, in itself, for delivery of chemotherapeutic agents to tumours, does not imply an increase in the latter. We found that angiotensin II increased tumour blood flow *relative* to various normal tissues but significantly decreased *absolute* flow. Other studies, in different tumour systems, have also shown a direct response of tumour blood vessels to vasoactive agents (Jirtle *et al.*, 1978, Weiss *et al.*, 1986, Tveit *et al.*, 1987).

All tested doses of angiotensin II reduced blood flow to the P22 tumour by a similar amount. These doses covered a range of perfusion pressures which suggests that angiotensin II increased tumour vascular resistance in a dose-dependent manner in order to overcome the tendency for blood flow to increase with increasing perfusion pressure. The results for tumour vascular resistance did show a trend towards a dose response but this was not statistically significant.

Our results for the effect of angiotensin II on blood flow heterogeneity are not readily comparable with other in the literature since those studies reported an overall increase, not decrease, in blood flow in their tumour systems (Hori *et al.*, 1985; Trotter *et al.*, 1991; Suzuki *et al.*, 1991). In the P22 tumour there was no difference in the overall intra-tumour heterogeneity of blood flow between control and angiotensin II-treated rats. This implies that angiotensin II is equally effective in reducing blood flow in the low flow regions as in the high flow regions when whole tumours are analysed. However, the reduction in blood flow at the highest perfusion pressure was due entirely to the response of the peripheral tumour regions. Blood flow to tumour sections cut from the tumour centre was unchanged. This suggests that there maybe some vascular collapse in the centre of the P22 tumour which the highest perfusion pressure could overcome. Vascular collapse may be more pronounced in the tumour centre than the periphery as a result of higher interstitial fluid pressure (Wiig *et al.*, 1982; Boucher *et al.*, 1990; Less *et al.*, 1991). Alternatively or, in addition, there may be a higher density of angiotensin II receptors at the periphery of these tumours than at the centre, possibly associated with a higher fraction of normal, incorporated vessels at the periphery than at the centre. The fact that large regions of very low flow, unconnected with necrosis, were observed in tumours from a minority of the angiotensin II-treated rats also suggests that there may be heterogeneous distribution of angiotensin II receptors in these tumours.

In summary,

(1) The P22 tumour was found to be directly responsive to the vasonconstricting effects of angiotensin II. The response was of the same order as that in skeletal muscle and kidney.

(2) The vasoconstriction outweighed the effect of an increase in perfusion pressure, induced by angiotensin II, resulting in a decrease in tumour blood flow.

(3) Overall, the intra-tumour heterogeneity of blood flow was not altered by angiotensin II, although, for the highest perfusion pressure studied, the reduction in overall tumour blood flow was due entirely to the response of the tumour periphery. In a minority of treated tumours, large areas of very low flow were observed. These regions were not related to necrosis.

(4) These results suggest that tumours other than the P22 could also respond directly to angiotensin II and other vasoactive agents. Analysis of inter- and intra-tumour heterogeneity of angiotensin II receptor number, determination of the ratio of normal to new blood vessels within tumours and measurement of physiological tumour parameters such as microvascular pressure and interstitial fluid pressure is a rational approach to finding a method for predicting tumour blood flow response.

We would like to thank Mr P. Russell and his staff for care of the animals. We would also like to thank Dr P. Carnochan for some free samples of $^{125}$I-IAP, Dr V. Cunningham for his blood flow programme and Dr D. Hirst for some useful discussion. This work was funded by the Cancer Research Campaign.

### References

ANDERSON, J.H., WILLMOTT, N., BESSENT, R., ANGERSON, W.J., KERR, D.J. & MCARDLE, C.S. (1991). Regional chemotherapy for inoperable renal carcinoma: a method of targeting therapeutic microspheres to tumour. *Br. J. Cancer*, **64**, 365–368.

BOUCHER, Y., BAXTER, L.T. & JAIN, R.K. (1990). Interstitial pressure gradients in tissue-isolated and subcutaneous tumors: implications for therapy. *Cancer Res.*, **50**, 4478–4484.

BURTON, M.A., GREY, B.N., SELF, G.W., HEGGIE, J.C. & TOWNSEND, P.S. (1985). Manipulation of experimental rat and rabbit tumor blood flow with angiotensin II. *Cancer Res.*, **45**, 5390–5393.

EKELUND, L. & LUNDERQVIST, A. (1974). Pharmacoangiography with angiotensin. *Radiology*, **110**, 533–540.

GOLDBERG, J.A., MURRAY, T., KERR, D.J., WILLMOTT, N., BESSENT, R.G., MCKILLOP, J.H. & MCARDLE, C.S. (1991). The use of angiotensin II as a potential method of targetting cytotoxic microspheres in patients with intrahepatic tumour. *Br. J. Cancer*, **63**, 308–310.

HEMINGWAY, D.M., COOKE, T.G., CHANG, D., GRIME, S.J. & JENKINS, S.A. (1991). The effects of intra-arterial vasoconstrictors on the distribution of a radiolabelled low molecular weight marker in an experimental model of liver tumour. *Br. J. Cancer*, **63**, 495–498.

HIRST, D.G., HIRST, V.K., SHAFFI, K.M., PRISE, V.E. & JOINER, B. (1991). The influence of vasoactive agents on the perfusion of tumours growing in three sites in the mouse. *Int. J. Radiat. Biol.*, **60**, 211–218.

HORI, K., SUZUKI, M., ABE, I., SAITO, S. & SATO, H. (1985). Increase in tumor vascular area due to increased blood flow by angiotensin II in rats. *JNCI*, **74**, 453–459.

HORI, K., SUZUKI, M., TANDA, S., SAITO, S., SHINOZAKI, M. & ZHANG, Q.H. (1991). Fluctuations in tumor blood flow under normotension and the effect of angiotensin II-induced hypertension. *Jpn. J. Cancer Res.*, **82**, 1309–1316.

HORSMAN, M.R., CHRISTENSEN, K.L. & OVERGAARD, J. (1989). Hydralazine-induced enhancement of hyperthermic damage in a C3H mammary carcinoma *in vivo*. *Int. J. Hyperthermia*, **5**, 123–136.

JIRTLE, R., CLIFTON, K.H. & RANKIN, J.H.G. (1978). Effects of several vasoactive drugs on the vascular resistance of MT-W9B tumors in W/Fu rats. *Cancer Res.*, **38**, 2385–2390.

KAPLAN, J.H. & BOOKSTEIN, J.J. (1972). Abdominal visceral pharmaco-angiography with angiotensin. *Radiology*, **103**, 79–83.

KETY, S.S. (1960). Theory of blood tissue exchange and its application to measurements of blood flow. *Methods Med. Res.*, **8**, 223–227.

KOBAYASHI, H., HASUDA, K., AOKI, K., TANIGUCHI, S. & BABA, T. (1990). Systemic chemotherapy in tumour-bearing rats using high-dose cis-diamminedichloroplatinum (ii) with low nephrotoxicity in combination with angiotensin II and sodium thiosulfate. *Int. J. Cancer*, **45**, 940–944.

KOBAYASHI, H., HASUDA, K., TANIGUCHI, S. & BABA, T. (1991). Therapeautic efficacy of two-route chemotherapy using cis-diamminedichloroplatinum (II) and its antidote, sodium thiosulfate, combined with the angiotensin-II-induced hypertension method in a rat uterine tumor. *Int. J. Cancer*, **47**, 893–898.

LESS, J.R., POSSNER, M.C., BOUCHER, Y., WOLMARK, N. & JAIN, R.K. (1991). Elevated interstitial fluid pressure in human tumors. *Pro. Am. Assoc. Cancer Res.*, **32**, 59.

NOGUCHI, S., MIYAUCHI, K., NISHIZAWA, Y., SASAKI, Y., IMAOKO, S., IWANAGA, T., KOYAMA, H. & TERASAW, T. (1988). Augmentation of anti-cancer effect with angiotensin II in intra-arterial infusion chemotherapy for breast carcinoma. *Cancer*, **62**, 467–473.

SASAKI, Y., IMAOKA, S., HASEGAWA, Y., NAKANO, S., ISHIKAWA, O., OHIGASHI, H., TANIGUCHI, K., KAYAMA, H., IWANAGA, T. & TERASAWA, T. (1985). Changes in distribution of hepatic blood flow induced by intra-arterial infusion of angiotensin II in human hepatic cancer. Cancer, **55**, 311–316.

SENSKY, P.L., PRISE, V.E., TOZER, G.M., SHAFFI, K.M. & HIRST, D.G. (1992). Resistance to flow through tissue-isolated tumours located in two different sites. Submitted to *Br. J. Cancer*.

SUZUKI, M., HORI, K., ABE, I., SAITO, S. & SATO, H. (1981). A new approach to cancer chemotherapy: selective enhancement of tumor blood flow with angiotensin II. *JNCI*, **67**, 663–669.

SUZUKI, N., SAKO, K. & YONEMASU, Y. (1991). Effects of induced hypertension on blood flow and capillary permeability in rats with experimental brain tumors. *J. Neurooncol.*, **10**, 213–218.

TAKEMATSU, H., TOMITA, Y. & KATO, T. (1985). Angiotensin-induced hypertension and chemotherapy for multiple lesions of malignant melanoma. *Brit. J. Dermatol.*, **113**, 463–465.

TANDA, S., HORI, K., SAITO, S., SHINOZAKI, M., ZHANG, Q.H. & SUZUKI, M. (1991). Comparison of the effects of intravenously bolus-administered endothelin-1 and infused angiotensin II on the subcutaneous tumor blood flow in anaethetized rats. *Jpn. J. Cancer Res.*, **82**, 958–963.

TOKUDA, K., ABE, H., AIDA, T., SUGIMOTO, S. & KANEKO, S. (1990). Modification of tumor blood flow and enhancement of therapeutic effect of ACNU on experimental rat gliomas with angiotensin II. *J. Neurooncol.*, **8**, 205–212.

TOZER, G.M., MAXWELL, R.J., GRIFFITHS, J.R. & PHAM. P. (1990*a*). Modification of the $^{31}$P magnetic resonance spectra of a rat tumour using vasodilators and its relationship to hypotension. *Br. J. Cancer*, **62**, 553–560.

TOZER, G.M. & MORRIS, C. (1990*b*). Blood flow and blood volume in a transplanted rat fibrosarcoma: comparison with various normal tissues. *Radiotherapy and Oncology*, **17**, 153–166.

TOZER, G.M., LEWIS, S., MICHALOWSKI, A. & ABER, V. (1990*c*). The relationship between regional variations in blood flow and histology in a transplanted rat fibrosarcoma. *Br. J. Cancer*, **61**, 250–257.

TROTTER, M.J. & CHAPLIN, D.J. (1991). Chemical modifiers of tumor blood flow. In *Tumor Blood Supply and Metabolic Microenvironment*. Gustav Fischer Verlag: Stuttgart, New York, Eds. P. Vaupel & R.K. Jain, pp. 65–85.

TROTTER, M.J., CHAPLIN, D.J. & OLIVE, P.L. (1991). Effect of angiotensin II on intermittent tumour blood flow and acute hypoxia in the murine SCCVII carcinoma. *Eur. J. Cancer*, **27**, 887–893.

TVEIT, K., WEISS, L., LUNDSTAM, S. & HULTBORN, R. (1987). Perfusion characteristics and norepinephrine reactivity of human renal carcinoma. *Cancer Res.*, **47**, 4709–4713.

VAUPEL, P. (1975). Interrelationship between mean arterial blood pressure, blood flow and vascular resistance in solid tumor tissue of DS-carcinosarcoma. *Experentia*, **31**, 587.

WEISS, L., TVEIT, E., JANSSON, I. & HULTBORN, R. (1986). Vascular reactivity to norepinephrine of 7, 12-dimethylbenz(a)anthracene-induced rat mammary tumors and normal tissue as studied *in vitro*. *Cancer Res.*, **46**, 3254–3257.

WIIG, H., TVEIT, E., HULTBORN, R., REED, R.K. & WEISS, W. (1982). Interstitial fluid pressure in DMBA-induced rat mammary tumours. *Scand. J. Clin. Lab. Invest.*, **42**, 159–164.